# Dry EEG measurement of P3 to evaluate cognitive load during sitting, standing, and walking

**DOI:** 10.1371/journal.pone.0287885

**Published:** 2023-07-06

**Authors:** Margaret M. Swerdloff, Levi J. Hargrove

**Affiliations:** 1 Department of Biomedical Engineering, Northwestern University, Chicago, Illinois, United States of America; 2 Regenstein Center for Bionic Medicine, Shirley Ryan AbilityLab, Chicago, Illinois, United States of America; Universiti Tunku Abdul Rahman, MALAYSIA

## Abstract

Combining brain imaging with dual-task paradigms provides a quantitative, direct metric of cognitive load that is agnostic to the motor task. This work aimed to quantitatively assess cognitive load during activities of daily living–sitting, standing, and walking–using a commercial dry encephalography headset. We recorded participants’ brain activity while engaging in a stimulus paradigm that elicited event-related potentials. The stimulus paradigm consisted of an auditory oddball task in which participants had to report the number of oddball tones that were heard during each motor task. We extracted the P3 event-related potential, which is inversely proportional to cognitive load, from EEG signals in each condition. Our main findings showed that P3 was significantly lower during walking compared to sitting (*p* = .039), suggesting that cognitive load was higher during walking compared to the other activities. There were no significant differences in P3 between sitting and standing. Head motion did not have a significant impact on the measurement of cognitive load. This work validates the use of a commercial dry-EEG headset for measuring cognitive load across different motor tasks. The ability to accurately measure cognitive load in dynamic activities opens new avenues for exploring cognitive-motor interactions in individuals with and without motor impairments. This work highlights the potential of dry EEG for measuring cognitive load in naturalistic settings.

## Introduction

Changes in performance during dual-tasking can help provide a better understanding of the cognitive requirements of motor tasks. Dual-task methods pair a cognitive task (e.g., serial subtraction) with a motor-based secondary task (e.g., walking). Dual-tasking causes cognitive-motor interference, which influences performance on either the motor task (motor control cost) or the cognitive task (cognitive cost), depending on the severity and intensity of the task [1]. Cognitive motor-interference stems from the limited processing resources of the brain. Specifically, cognitive responses to external stimuli are reduced during dual-task scenarios when there is competition between cognitive and motor resources [2]. This ‘bottlenecking’ of cognitive and motor processes can be compensated for through changes in gait biomechanics that preserve the physical stability of walking and decrease the likelihood of falling, or through decreasing attention towards cognitive tasks.

Cognitive-motor interference is demonstrated when a person’s general walking ability is compromised, such as in elderly individuals [3] and individuals with lower-limb loss [4]. Compensatory strategies, such as wider stance and more time spent in the double stance phase of gait, are employed to increase stability when cognitive limits have been reached. For example, Pruziner *et al*. found individuals with lower limb amputation exhibited a wider base of support and more stable gait patterns when assigned a cognitive task compared to walking without any additional task [5]. Similarly, Al-Yahya *et al*. found that dual-tasking, increased age, and changes in mental state were found to reduce gait speed during walking within a range of impaired and unimpaired populations [1].

In persons without motor-impairments or during motor activities other than walking, gait preserving compensatory strategies may be difficult to measure or not present in typical gait performance measures such as gait speed. However, in this case, cognitive costs may still be present and measurable from the brain’s underlying cortical dynamics. Non-invasive brain imaging techniques such as electroencephalography (EEG) offer insight into the cognitive processing during taxing cognitive events, even in the absence of motor control costs. EEG can also be used in mobile and stationary settings and provide a task-agnostic modality for directly assessing cognitive load. In De Sanctis *et al*., no motor control costs were seen in terms of the participants’ ability to complete a cognitive task (a Go/No Go response inhibition task), whereas robust differences in cognitive cost, namely in event-related potential components, were seen between the motor tasks (sitting and walking) [6].

Defining the concept of cognitive load has been a challenge particularly in the field of wearable assistive devices. Cognitive load has been defined as “the demands placed by a task on the mental resources of the individual to process task-related information” [7]. There are a number of factors that influence cognitive load; notably, emotional fatigue and neural fatigue have important contributions to overall cognitive load in relation to amputation [8]. Furthermore, the intrinsic difficulty of the task performed depends on the subjective experience of the user [9]. Thus, two people may experience the same task with varying levels of cognitive load. This points to the need for a quantitative measure of cognitive load that is objective and can be applied widely in populations who use wearable assistive devices.

Event-related potentials (ERPs) provide a quantitative measure of cognitive load from brain responses to stimuli such as auditory, visual, or tactile cues. Stimulus paradigms used to elicit ERPs commonly include the oddball paradigm, in which a participant attends to a train of target and non-target stimuli. The participant is asked to ignore the frequently-occurring non-target stimuli and keep a mental count of the target (oddball) stimuli [10]. The amplitude of the brain response, or the EEG signal at the time of stimulus onset, reflects the amount of cognitive processing that takes place when the stimulus is perceived. Due to the limited processing resources of the brain, the cognitive response to external stimuli is reduced during dual-task scenarios when more cognitive resources are required [2]. The P3 potential is the third positive peak in the ERP, found at approximately 300 ms after stimulus presentation [2]. The P3 potential is thought to represent context-dependent processing of external stimuli [2], and its amplitude has an inverse relationship to cognitive load [10].

Advances in wireless electrode technology have recently allowed EEG measurement during movement in unconstrained environments such as table tennis [11], jogging [12], and cycling [13]. Some studies do not measure or explicitly account for the possible degradation of EEG signal quality due to movement-related artifacts [6]. Other works have explored the removal of movement-related artifact with mixed results. Some groups suggest that walking-related motion artifacts are negligible for gait speeds below 4.5 km/h [14]. However, others claim that motion artifacts do impact signal from gel-based EEG during walking and that motion related artifacts are not removable using traditional signal processing methods [15]. These results point toward the continued need for evaluating the impact of movement-related artifacts on EEG signal quality.

Factors that impact the post processing signal quality are the artifact removal methods and parameters, the EEG hardware (wet, dry, or gel), and the type of analysis performed on the EEG signal. Despite this variety of factors, most studies that looked at the impact of movement-related artifacts used continuous and spectral analysis of gel-based EEG [14] rather than ERP from dry EEG. A limitation of gel-based EEG is that it requires the application of conductive paste. Application and cleanup of the conductive paste can be uncomfortable for users [16]. Throughout the duration of the session, gel may need to be reapplied to avoid poor readings. Furthermore, applying excessive amounts of gel can result in short circuiting between sensors [17]. An advantage of dry EEG is reduced cross-talk between electrodes [16]. Dry EEG provides increased participant comfort in that there is no need for gel application, skin preparation that could lead to bacterial infection, or post-record scalp cleaning [16]. Dry EEG also allows for faster setup time [18] and rapid self-application, which could make at home-trials possible [19]. Faster setup of the EEG may allow additional time for experimentation, including potentially evaluating and adjusting the assistive device which could improve the quality of the study. Although previous work has measured differences between EEG systems [20], this work uses a dry EEG headset with digital and physical attributes that are not necessarily unique to this headset but contribute to its ability to tolerate movement artifact. The physical equipment setup includes spring-loaded electrodes with a head strap. These attributes may consequently lead to a more robust signal to noise ratio [21] and potentially negligible effect of head movement artifact on the EEG signal [22]. While no other authors have used this particular dry EEG system (DSI-7) to evaluate ERP during walking, ERP during outdoor walking has been evaluated using at least one other dry EEG system [23].

Evaluating the ability of populations with motor impairments to safely navigate inside and outside the home includes the assessment of tasks associated with many activities [24–26]. Three critical tasks that test the range of activities in ecologically valid settings include sitting, standing, and walking [24]. There have been a number of studies that found a reduction in P3 during walking compared to sitting or standing [6, 27–30]. However, all previous studies that compared ERP in sitting, standing to that of walking have used a gel-based EEG, not a dry EEG. For example, Protzak *et al*. recorded ERP in sitting, standing, and walking in younger adults (and compared them to older adults) with gel-based EEG [27] rather than dry EEG. Advantages of dry EEG include faster setup time [18] and rapid self-application [19], which may allow future work in clinical populations participating in at-home trials. Due to improvements in its digital and physical attributes, the DSI-7 may provide superior artifact removal compared to other systems [21, 22]. However, as dry EEG systems are prone to possible motion artifacts, there is a need to evaluate possible movement-related artifacts with the DSI-7 (Wearable Sensing).

The goal of the present study was to implement an auditory stimuli paradigm with a dry EEG headset during multiple mobile and stationary tasks (i.e., walking, standing, and sitting) and to evaluate the impact of possible motion-related artifacts in this dry EEG system’s signal. To our knowledge, this study is the first to compare P3 across the tasks of sitting, standing, and walking using dry EEG with an auditory oddball paradigm. The main contribution of this work is the validation of a commercial dry-EEG headset for measuring cognitive load across different motor tasks. This validation expands the potential use of dry EEG systems in cognitive neuroscience, particularly for studies conducted in naturalistic settings or involving individuals with motor impairments. This work also examines the possible impact of motion on the P3 ERP component during walking, the most motion-inducing condition of the three. We hypothesized that the cognitive requirements of walking will result in a reduction of the P3 amplitude compared to sitting and standing, as shown in studies using wet EEG and a visual cognitive task [27]. We also hypothesized that standing will be more cognitively demanding than sitting, as suggested by prior studies using dual-tasks [31] and P3 amplitude [27].

## Methods

All experiments were approved by the ethics committee of the Institutional Review Board of Northwestern University (STU00208516). Experiments were carried out in accordance with the Institutional Review Board guidelines and regulations. All subjects provided written informed consent before participating in the study. Ten participants (5 female, 5 male, mean age 22 ± 3 years, age range 20–29 years) were recruited for this study. Results from one subject were excluded from the analysis due to cardioballistic artifacts in the EEG signal, thereby leaving data from nine participants in our final analysis.

Participants completed three sessions each for three conditions: sitting, standing, and walking. The conditions were completed in a randomized, counter-balanced order so that each session consisted of one of each condition. Our pilot studies indicated that participants became too fatigued with long sessions of 15 minutes each. Thus, our current study separated each condition into three separate 5-minute tasks, with each set of three tasks followed by a 5-minute break. The total session time was approximately one hour. Stimuli were delivered in a randomized order and included 90% target (standard) tones and 10% non-target (oddball) tones (270 non-target trials and 30 target trials per task), according to a two-tone auditory oddball paradigm [32]. Auditory stimuli were applied at random intervals between 675 and 1365 ms, which was chosen based on 1000 ms intervals ± 365 ms jitter, in order to include 30 target tones within each task of approximately 5 minutes duration. Oddball stimuli were infrequently occurring high-pitched tones at 1200 Hz and standard stimuli were frequently occurring non-target low-pitched tones at 900 Hz. Stimuli were played by a microcontroller (Arduino) with an audio wave shield to wired earbuds. Before beginning each experiment, participants were allowed to adjust the volume on the stimulus delivery and verified that they could clearly distinguish between the two types of tones.

**Fig 1:**
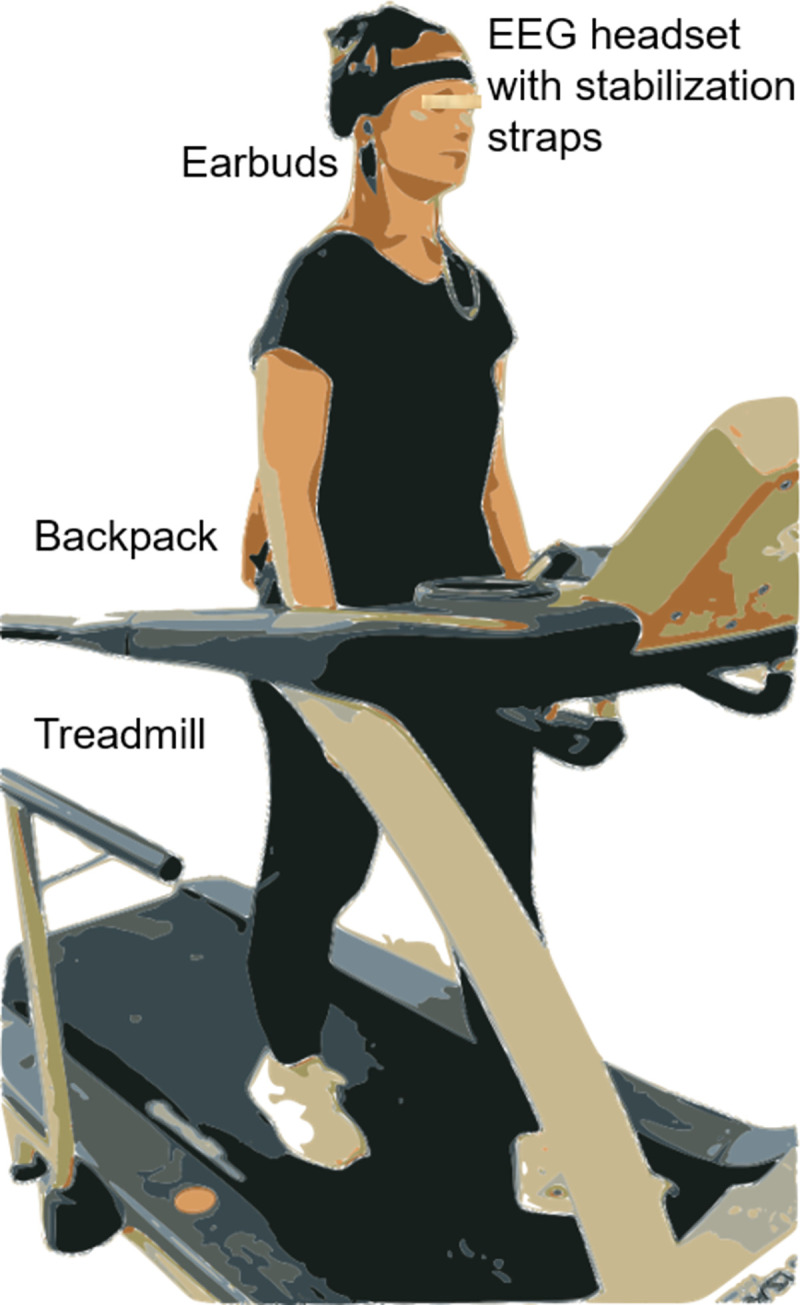
A participant during treadmill walking wearing an EEG headset with stabilization straps, headphones for listening to auditory oddball stimuli, and backpack containing stimulus-synchronization equipment (Trigger Hub, Wearable Sensing).

The audio signal was simultaneously delivered to the headphones and the stimulus Trigger Hub (Wearable Sensing) which time-stamped the timing onset for each stimuli. Each time an auditory tone was played, the Trigger Hub created a trigger that was synchronized with the EEG signal. Participants wore the EEG cap along with a lightweight backpack containing the Trigger Hub and Arduino microcontroller (Fig 1).

**Fig 2:**
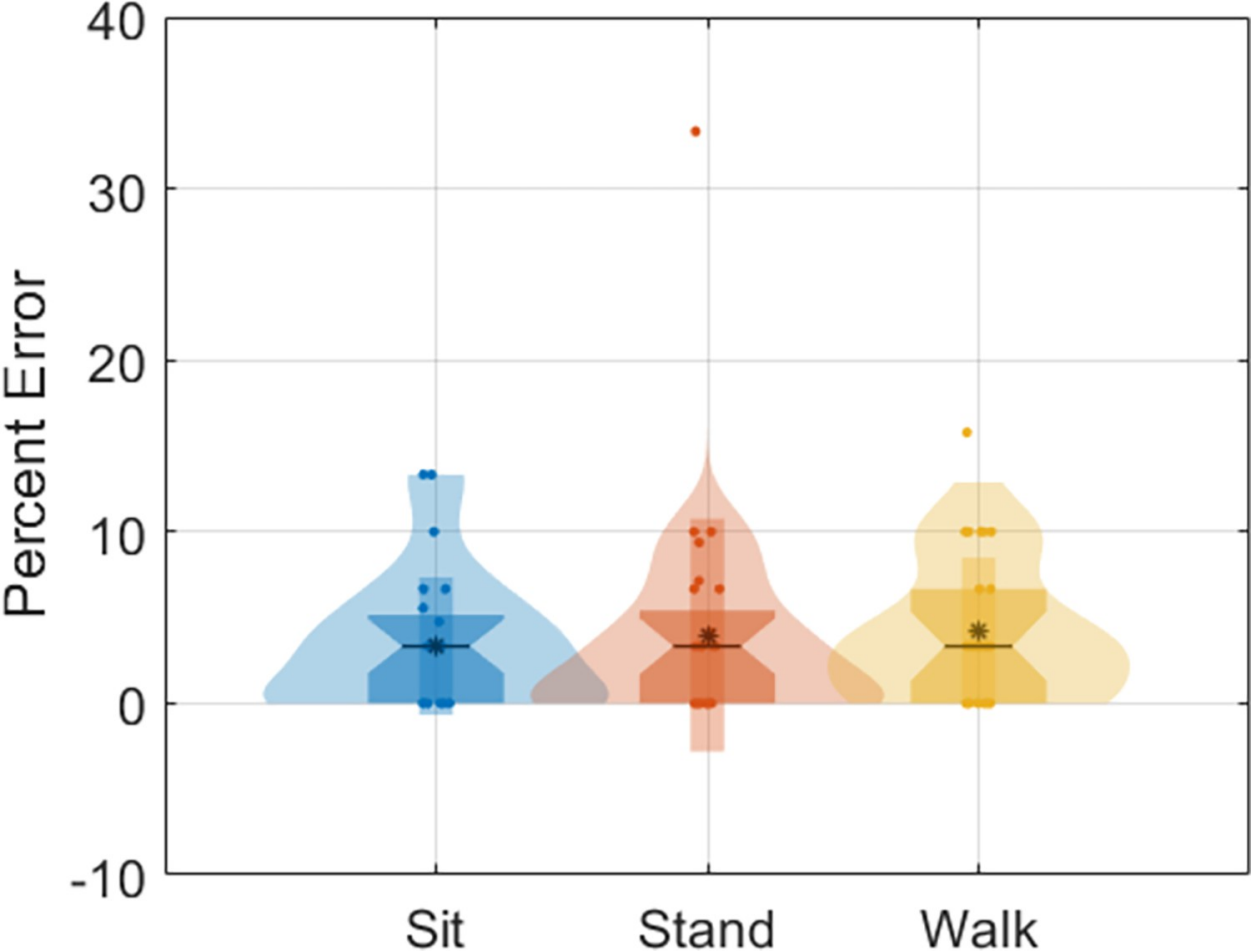
Counting task error during oddball stimulus task. All raw data points are shown on top of kernel density plots and boxplots (1st thru 3rd quartiles). Medians are denoted by horizontal lines between 95^th^ percentile notches and mean values are denoted by asterisks. No significance differences were found for task error (*p* = .86).

Participants were asked to count the number of target tones they heard in each session. Differences in reaction time between motor tasks were not measured as there was no physical button press. This decision was made to reduce confounding the P3 response with the motor activity associated with pushing a button [33]. Instead, participants kept a mental count of the target tones heard throughout the task, and the total number of target tones heard by each participant was recorded and compared against the actual number, which was documented as the task error. Task error was measured as the difference between the number of tones actually played and the number that was reported to be heard by the participant (Fig 2). A two-way 3x3 ANOVA was calculated for task error. The factors that were included were session and condition. The significance level was set to *p* < 0.05.

**Fig 3:**
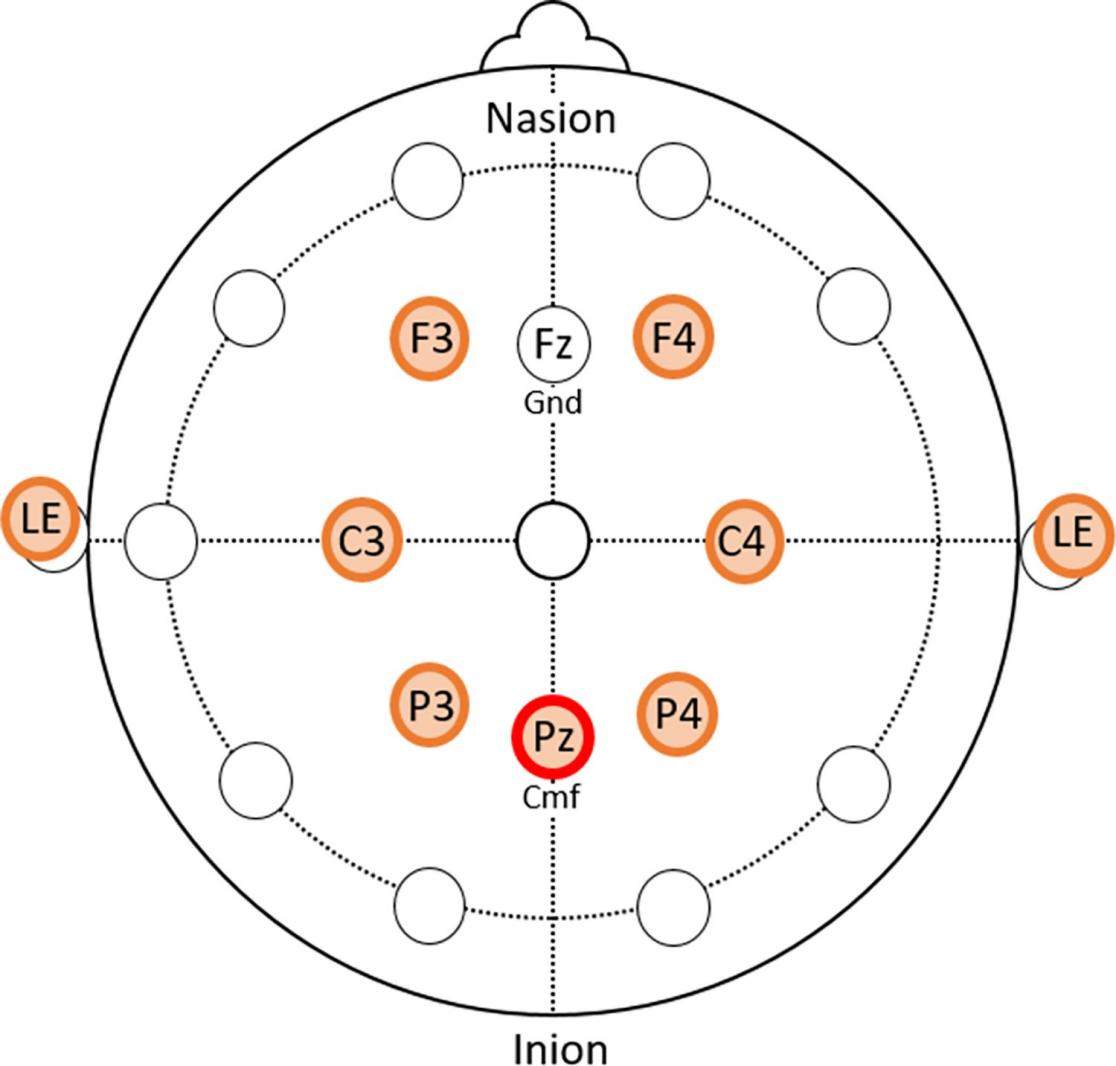
Scalp electrode placement. Pz (circled in red) is the location of the common mode follower.

EEG signal was recorded using a DSI-7 (Wearable Sensing), a wireless headset with seven dry electrodes located on the scalp at F3, F4, C3, C4, P3, Pz, and P4 (Fig 3). The ground electrode was located at Fz. Linked ears (LE) reference electrodes were placed on both earlobes. Signals were recorded at 300 Hz through an impedance amplifier. The impedance was monitored to ensure that it was below 1 MΩ before starting the experiment. Participants wore a stabilization strap with Velcro straps to secure the EEG cap. A 3-axis accelerometer located inside the EEG cap measured head movement in each condition.

**Fig 4:**
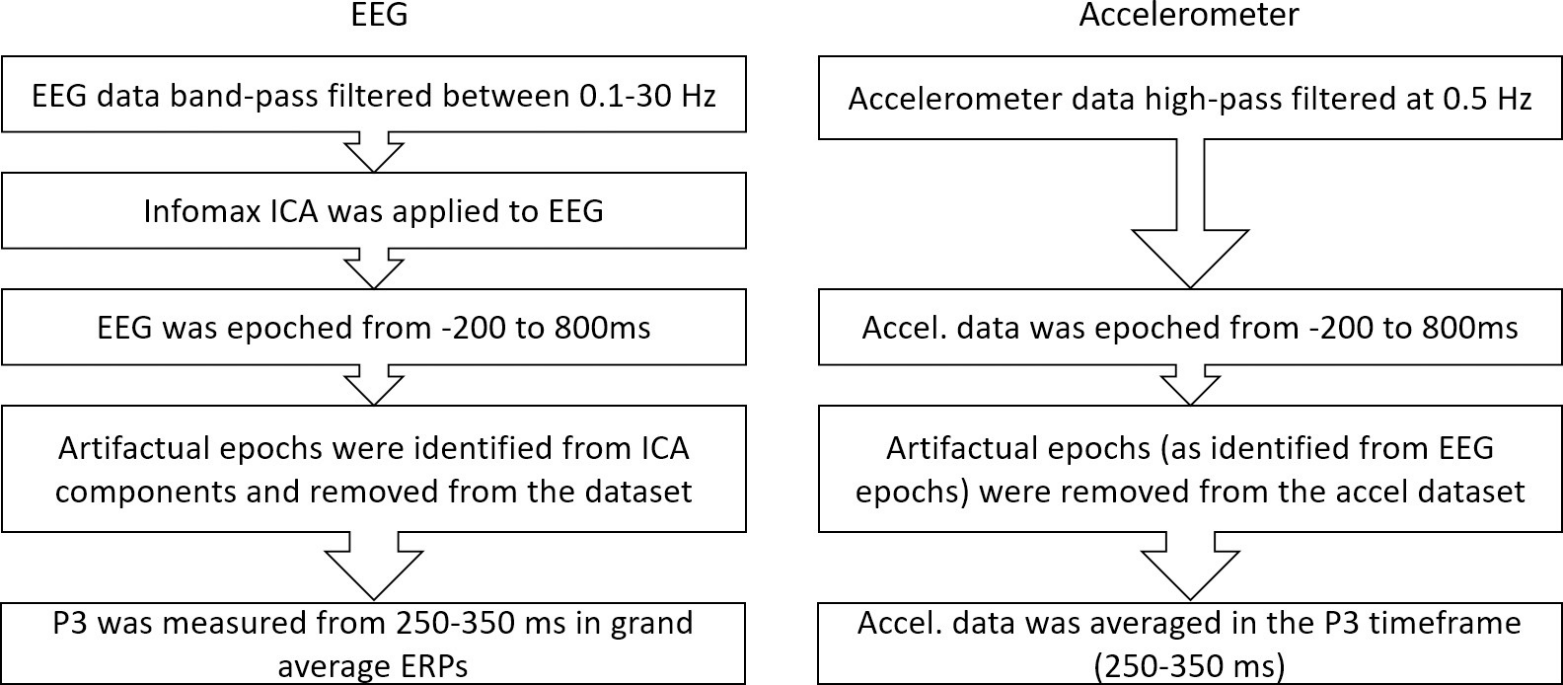
Outline of data analysis pipeline for EEG and accelerometer signals.

A flowchart outlines the following data pipeline (Fig 4). Raw EEG data were band-pass filtered between 0.1 Hz to 30 Hz through a zero-phase 4th order Butterworth filter offline, similar to previous ERP studies [34]. Infomax Independent Component Analysis (ICA) was applied to the continuous filtered data to separate neural from non-neural components with custom scripts written in MATLAB using the plug-in EEGLAB [35], along with built-in functions from ERPLAB [36]. All trials (from continuous data) were used for the ICA signal decomposition. The ICA components were used for the artifact identification process as described in Swerdloff *et al*. [18]. The small number of electrodes used here is not sufficient for robust source decomposition via ICA; thus, in lieu of source decomposition, the ICA process was used in the identification of contaminated epochs from ICA components containing eye-movement related artifacts. No independent components from the ICA decomposition were removed from the dataset.

To create epochs, EEG data was sectioned and aligned to the onset of each stimuli, from -200 ms prior to stimulus onset to 800 ms post stimulus onset. Epochs contaminated by artifacts (eye-blink, muscular and cardiovascular artifacts, etc.) were identified from the ICA components according to standard and previously-used parameters [18, 37]. Rejection criteria included abnormal values (i.e., those outside the range of -25 to 25 *μ*V in the pre-stimulus period and -75 to 75 *μ*V post stimulus period), strong linear trends (maximum slope of 50 and r-squared up to 0.3), abnormal joint probabilities (single-component and all component probabilities of up to 5), strong kurtosis (distributions with kurtosis up to 5), and abnormal spectral properties (i.e., those outside the range of -50 to 50 dB between 0 to 2 Hz and from -100 to 25 dB between 20 to 40 Hz).

Grand average ERP were generated by aligning the onset of the stimuli and averaging across trials. The P3 timeframe was chosen as 250–350 ms based the peak P3 amplitude from the grand average waveform from Target trials across all conditions (P3 peak amplitude latency was 320 ms averaging all sitting, standing, and walking trials). After removing artifact trials, the average acceptance ratio was 83% for sitting, 84% for standing, and 64% for walking. There were at least 37 artifact-free trials per condition per subject. Although a classic subject-exclusionary approach would require approximately 20 trials or more per subject [38], a linear mixed effects model can include all subjects with least one trial because the model does not assume equal numbers of trials across subjects [39]. Thus, instead of excluding subjects having below a certain number of non-artifact trials, we used a linear mixed effects model as a conservative method to determine statistical significance. Statistically significant model estimates indicated a statistical difference in that predictor variable from the baseline (i.e., sitting) [39]. Accelerometer signals were high-pass filtered at 0.5 Hz, segmented with the same timeframe as the EEG data, and aligned to stimuli onset [13]. The magnitude of the accelerometer RMS was taken to represent the head motion for each trial and averaged into eight bins to maintain consistency with previous literature which measured the impact of motion artifacts on ERP during cycling [13].

## Results

### Oddball auditory task applied during sitting, standing, and walking

Able-bodied participants performed a series of three activities: sitting, standing still, and walking on a treadmill, while wearing a dry EEG headset and associated stimulus synchronization equipment contained within a lightweight backpack (Fig 1). Oddball counting errors are shown for all conditions (Fig 2). Participants were able to complete the auditory oddball counting task with less than 8% task error. No significant differences in task error were found across conditions or sessions (*p* = .86), indicating that participants were able to complete the auditory cognitive task equally as well, on average, for any of the conditions or sessions.

### ERP differentiates walking from that of sitting and standing

**Fig 5:**
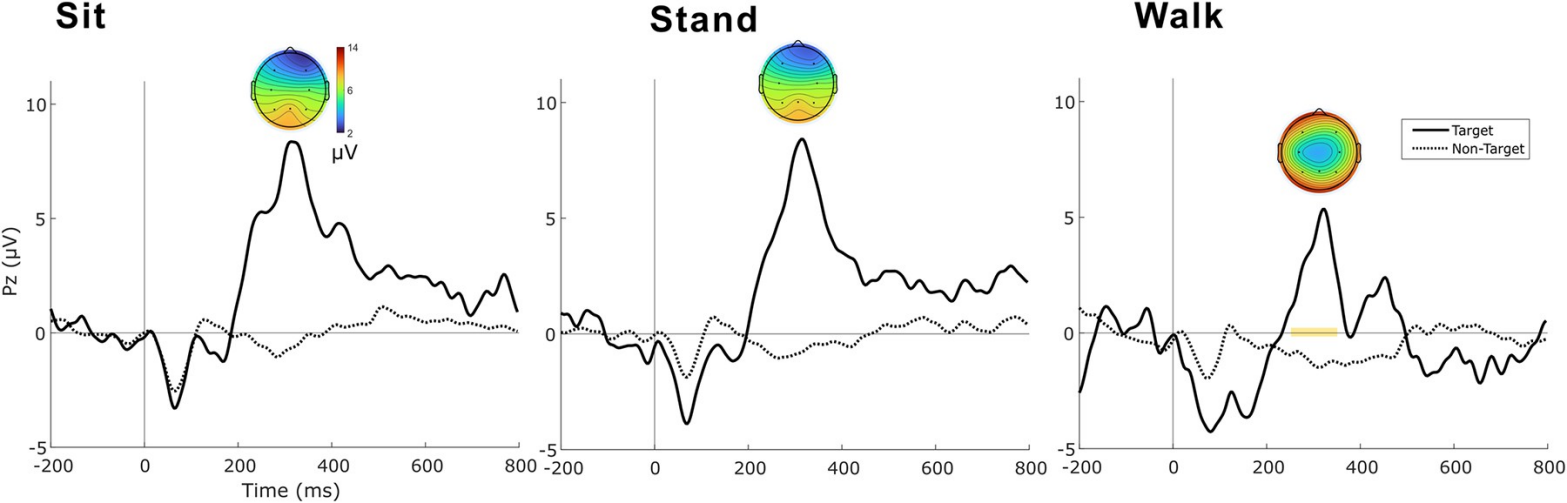
Grand average ERP from all conditions (sit, stand, and walk) for oddball (target) and frequently-occurring (non-target) tones, combined for all sessions. Yellow shading indicates significant difference in average voltage in the P3 interval (250 ms to 350 ms) during the walk condition compared to the sitting condition (*p* = .039). Insets show average brain activity across the entire scalp during the P3 timeframe (250 to 350 ms) for target stimuli.

The grand average ERP taken from the central parietal electrode (Pz, Fig 3) across all three conditions for both target and non-target stimuli is shown in Fig 5. Non-target ERP does not show any deflections, suggesting that motor-related cognitive activity did not impact cognitive responses to stimuli. In contrast to non-target ERP, the ERP for target stimuli showed a positive deflection at around 320 ms (P3) in all conditions. Average brain activity is also shown for electrodes across the scalp at the P3 timeframe (250 to 350 ms) for target stimuli in each condition (Fig 5, insets).

The ERP responses during the P3 timeframe were compared across all trials and conditions. A linear mixed effects model accounting for individual subject differences indicated that the average voltage during the P3 timeframe was significantly lower for walking compared to sitting (*p* = .039). This result suggests that cognitive load was greater during walking than during sitting.

### Head motion does not impact ERP during walking

**Fig 6:**
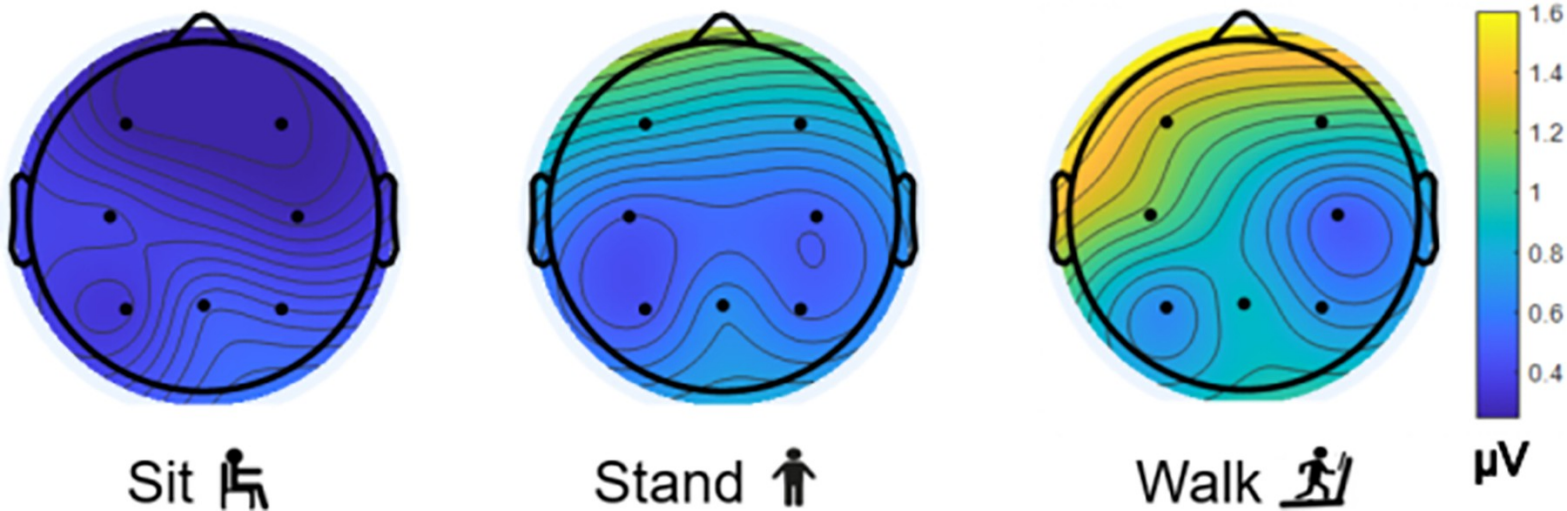
Scalp plots showing median RMS voltage during baseline periods of no stimulus presentation (i.e., from -200 to 0 ms).

**Fig 7:**
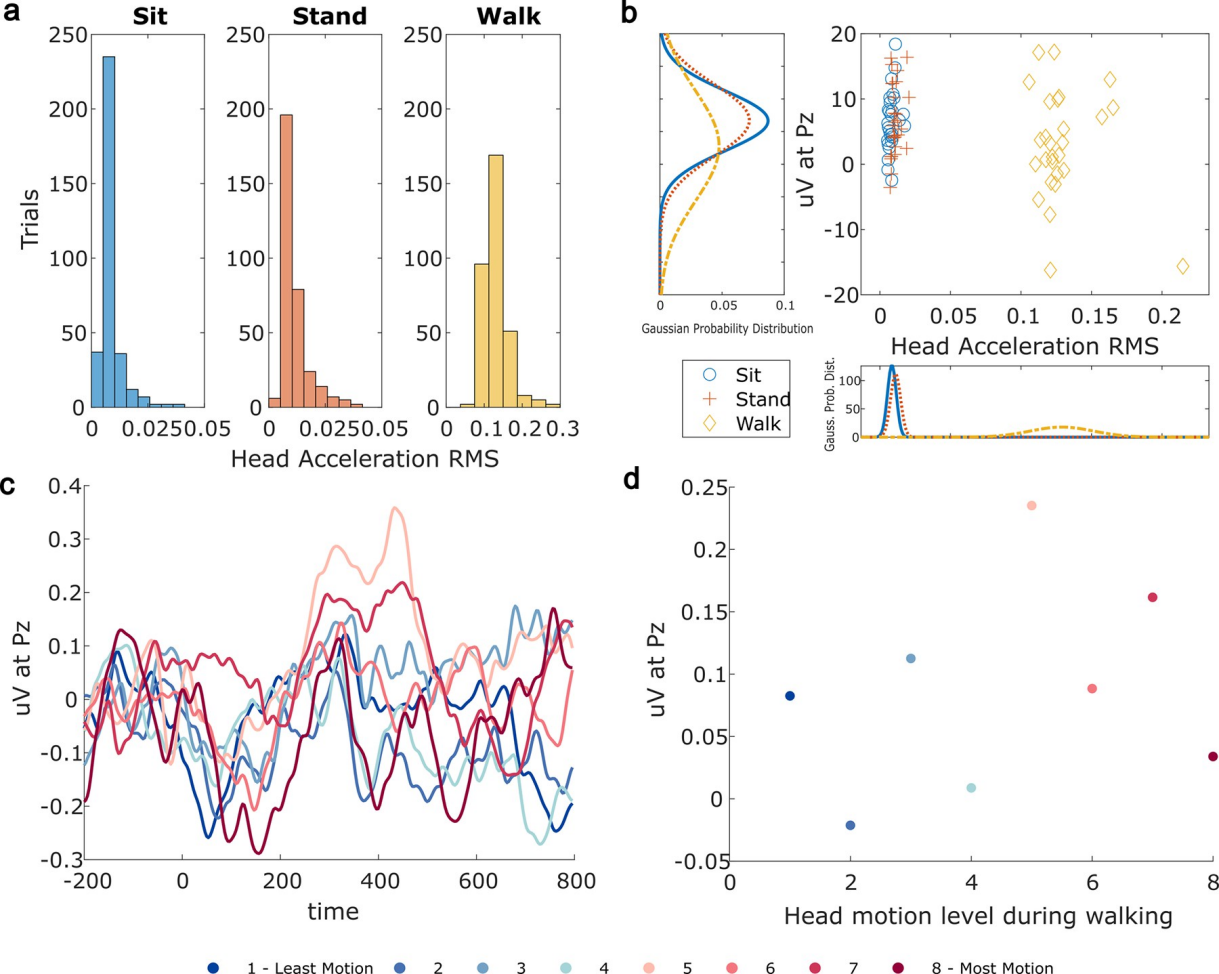
(A) Histogram of accelerometer RMS magnitude for each condition (sit, stand, and walk). (B) Mean P3 voltage at Pz plotted against the associated motion RMS vector for each subject in each condition. Axes show the Gaussian probability distributions for each condition. Colors correspond to those presented in A. (C) Averaged ERP for eight levels of head motion during walking. (D) Mean P3 voltage from ERP in C plotted against motion level, with each head motion level represented by a different color. Colors correspond to those presented in C.

Baseline EEG (RMS voltage) during the time prior to stimuli presentation showed the highest overall magnitude of the signal and had the most variation in EEG signal during walking as compared to sitting and standing (Fig 6). As such, the impact of motion on the EEG signal during walking was also analyzed. Fig 7A shows the distribution of motion across all trials in the sit, stand, and walk conditions. The mean and standard deviation of the RMS magnitude of the acceleration vector was greater during the walking condition as compared to that of the sitting and standing conditions. This was expected due to the increased motion of the head during walking. Fig 7B shows the average voltage during the P3 timeframe at the Pz electrode as a function of the motion during the corresponding P3 timeframe for each condition. The variation in motion (Fig 7B) was not correlated to voltage during the P3 timeframe for any condition (*r* < .5). Gaussian probability distributions (Fig 7 and Table 1) show that the mean P3 was lower for walking (*μ* = 2.809 *μ*V) compared to sitting (*μ* = 6.651 *μ*V) and standing (*μ* = 6.733 *μ*V) and the variability in the walk condition (*σ* = 8.39 *μ*V) was greater than for sit (*σ* = 4.58 *μ*V) and stand (*σ* = 5.54 *μ*V). To account for individual differences, the correlation was calculated for individual subjects. No individual trends were found (*r* < .5), suggesting that head motion did not impact the P3 responses.

**Table 1:** Estimated Gaussian parameters for RMS head motion and average P3 for each condition. (Summary of data shown in Fig 7B).

Condition	RMS Head Motion	Average P3 (*μ*V)
	mean (SD)	mean (SD)
Sit	.0085 (.002)	6.651 (4.58)
Stand	.0111 (.004)	6.733 (5.54)
Walk	.1209 (.022)	2.809 (8.39)

To further investigate the impact of motion on the P3 response, all walking trials were sorted according to their corresponding RMS magnitude from the accelerometer and binned according to eight motion levels, to get ERP for each motion level as done by Zink *et al*. [13]. Average ERP for each motion level is shown in Fig 7C. The average voltage in the P3 timeframe was plotted against the motion level during walking, as shown in Fig 7D. The average P3 voltage as a function of motion level during walking did not yield a significant trend (*r* = .24, *p* = .125).

## Discussion

Cognitive requirements that are known to impact activities of daily living in both healthy individuals and those with motor impairments have been difficult to measure due to methodological limitations. EEG provides a possible method to directly measure the ease of completing a task with high temporal resolution. This study is the first to use dry EEG to measure the cognitive load of three tasks: sitting, standing, and walking. The P3 response found in this study was lowest during walking, indicating that walking was the most cognitively burdensome task. These results support those of prior studies, including those that have compared the P3 responses during walking and sitting [5, 23, 27, 40, 41].

While our results indicate that walking had the lowest P3 amplitude compared to that of sitting (Fig 2), we found similar P3 amplitudes for both the sitting and standing tasks. As there was no significant differences between sitting and standing, and the goal was not to test the difference between walking and standing, we did not test for a possible significant difference between walking and standing. This finding contrasted with at least one other study which compared the cognitive load of sitting and standing: a dual-task study that found slower reaction times during standing compared to sitting [42]. While not significantly different, Protzak *et al*. [27] also showed higher cognitive load for standing compared to sitting. However, the visual task used in Protzak *et al*. [27] was different from that of our current study. Our work used an auditory oddball task that is easier to administer in unconstrained environments compared to visual stimuli, which may be the reason for this difference.

A result of this study is that the auditory task did not distinguish between the cognitive load of sitting and standing. However, this could be due to the low sample size, which is a limitation of this study. Alternatively, tasks that are nearly equally easy, as in the case of sitting and standing for able-bodied individuals, are not expected to yield differences in P3 unless the cognitive task is difficult enough. In contrast to the auditory task used in the current study, the visual task used by Protzak *et al*. [27] was able to distinguish the cognitive load between sitting and standing in healthy participants of similar age to those in the current study. This may have been because visual tasks are more difficult to complete during activities that require trunk support, as the balance required to maintain posture relies more on the visual system than the auditory system [43]. Thus, the auditory oddball task used here may have been not difficult enough for us to distinguish between the cognitive load of sitting and standing for the able-bodied individuals who participated in this study. The advantage of using auditory tasks is that they only require headphones, in comparison to visual tasks which require an environment outfitted with LEDs [27]. Future work may consider the use of a more difficult auditory task or a visual task in augmented reality to maintain the possibility of administering these tasks in unconstrained, outdoor environments.

While the auditory oddball task used in this study is appropriate for distinguishing the cognitive requirements of sitting compared to walking, it might be too simple to cause a change in the cognitive response shown in the ERP in populations without motor impairments when comparing sitting to standing. However, the lack of a difference in P3 between sitting and standing is an interesting finding in that it could inform future work with different tasks and populations with motor impairments. For example, we expect that individuals with poorer trunk support and balance would not find sitting and standing to be equally easy tasks and thus would have lower P3 amplitude for standing compared to sitting. Another area of interest is for lower-limb prosthesis users, as it is possible to use this paradigm to evaluate changes in cognitive load for users of different devices. For example, a microprocessor knee that provides stance support may be easier for a person to use while standing compared to a purely mechanical device that does not provide stance control.

Independent component analysis (ICA) is a widely used method that separates statistically independent sources in a continuous EEG signal, which can then be localized to specific brain regions using inverse head modeling. ICA is useful for obtaining source localization in high-density EEG, but reconstruction of neural activity with brain components is not yet optimal for low-density (e.g., below 32 channel) EEG compared to high-density EEG, although this may be attenuated using empirical mode decomposition [44]. Without using reconstruction, ICA is still useful for the identification of eye-blinks so that those segments can be removed from continuous EEG, even in low-density EEG [45]. Thus, we applied ICA to find components with ocular artifact, and instead of removing any possible non-brain components and then performing reconstruction, we simply use the component(s) representative of ocular artifact as part of the artifact rejection process. Some studies have examined the parameters used for optimal ICA during mobile experiments, such as filter cutoff [46], but these parameters may not be applicable for ICA as an artifact removal method instead of a means of source localization. Furthermore, to prevent attenuation of ERP waveforms, the analysis parameters that were chosen are those that are commonly used for ERP analysis (e.g., Tanner *et al*. [34]) instead of those commonly used for source-localization of mobile EEG (e.g., Klug *et al*. [46]), as source localization is not required for ERP analysis.

We noticed that the topography of the P3 component was different in the walking condition compared to that of sitting and standing (Fig 5). Typically, P3 is characterized by high amplitudes at the central/posterior electrodes and lower amplitudes at the lateral electrodes. However, our findings showed a somewhat altered topography, possibly due to noise in the lateral electrodes associated with walking. As our artifact correction methods were focused on the Pz electrode, this observation suggests that this method inadequately handled artifacts in lateral channels during mobile conditions. This is a limitation that highlights the complexities and challenges in mobile EEG data collection using dry electrodes. Walking, in particular, introduces additional movement artifacts, which may disproportionately affect certain channels, such as the lateral electrodes. It is important to note that our current artifact correction approach, while effective for central channels, may have limitations when applied to the lateral channels during movement. This could potentially result in less successful noise suppression in these channels, leading to the observed differences in P3 topography.

While previous works have demonstrated excellent signal to noise characteristics in wet EEG, only one previous study has used a dry EEG with ERP as we used in the current work [47]. Mathewson *et al*. required participants to attend to an auditory oddball task while sitting still [47]. Fortunately, there may be a lesser impact of movement-related artifacts on ERP compared to continuous EEG. Due to the averaging process involved in the methodology to obtain event-related potentials, there is a stronger likelihood that movement-related artifacts will be averaged out in ERP compared to continuous EEG, where there is no averaging processes [48]. For instance, Zink *et al*. examined the impact of motion on ERP recorded during biking in seated (non-moving), stationary (pedaling on a stationary bike), and moving (biking through a college campus) conditions and found no effect of movement artifacts on P3 amplitude [13]. Zink *et al*. also introduced using the RMS (root mean square) of accelerometer data as a measure of the amount of head motion per trial. This was compared to P3 amplitude and they demonstrated that head motion was not correlated to P3 amplitude. This study follows suit, but using a dry (i.e., gel-free) electrode EEG headset instead of a wet EEG.

Wireless, dry EEG provides additional benefits over wet or gel-based EEG methods. Dry EEG has a fast setup time (5–10 minutes, as in the current and previous study [18]), can be used in environments where gels are not allowed [49], and avoids the need for re-application of gel in prolonged experiments [19]. With the advent of EEG systems that are dry and mobile also comes the need to determine the effect of motion on EEG signal [50, 51]. Although not all dry electrode headsets provide the same level of signal quality [52], studies have found similar signal quality with dry electrodes compared to wet for certain systems [52, 53], including the DSI-7, the system used in this study, during seated [54] and dynamic testing environments [55]. Consequently, we analyze an additional modality that is greatly impacted by motion and can be measured during both conditions (i.e., acceleration).

Dry EEG headsets have not been widely used in dynamic environments due to a poor signal quality that varies greatly across different dry EEG systems [52, 53]. Although in one study by Oliveira *et al*., the authors reported that no data was usable (i.e., 100% of epochs were discarded) using their dry EEG system and removing epochs exceeding a threshold difference of 75 *μ*V from baseline. In contrast, the results of this study successfully yielded a suitable amount of clean epochs from dry EEG recorded during walking using the same standard threshold as in Oliveira *et al* [20]. Furthermore, our results indicate that movement did not affect EEG because head accelerometer RMS was not correlated with the signal from the Pz electrode located at the top of the head at the group nor at the individual levels (*r* < .5, Fig 7B). Additionally, motion levels were not correlated with ERP amplitudes at the group level (*r* = .24, Fig 7D). This difference in signal quality may be explained by differences in the dry EEG system technologies.

While there is proprietary information that may help explain the differences in dry EEG technology, the system used in the current study may provide superior signal quality in part due to its stabilization strap, spring-loaded electrodes, and common mode follower. Participants wore a Velcro strap wrapped around the forehead. This strap connected to the top of the cap, pulling it downwards to allow the spring-loaded electrodes to perform at their optimum pressure and maintain contact with the scalp.

This study provides a method for measuring cognitive load using a dry EEG interface that is robust enough to handle tasks of various dynamic movement artifact. Current methods in EEG allowed the measurement of EEG during mobile activities in ecologically relevant settings. Future work could use this methodology to understand the impact of cognitive load during dynamic activities. Variation in P3 across days, stress level, cognitive function, and levels of motor impairment for a range of dynamic tasks is important to identify and understand factors that influence cognitive load.

## Supporting information

S1 File Machine learning classification and SME analysis. (DOCX)
