# Supplementary material for: Dry EEG measurement of P3 to evaluate cognitive load during sitting, standing, and walking
Source: PLoS One. 2023 Jul 6;18(7):e0287885. doi: 10.1371/journal.pone.0287885 (PMC10325065; doi:10.1371/journal.pone.0287885)
Supplement: S1 File — (DOCX) [file pone.0287885.s001.docx]

We performed a machine learning based analysis to verify that the ability to recognize target from non-target tones was achievable between sitting and walking. All records from all subjects were used in this analysis. To train the machine learning model, the following steps were taken:

1. The responses from all sitting trials were evaluated from stimulus onset until 400 ms after the stimulus, the timeframe that includes the P3 response waveform.
2. These were grouped into two categories: target trials, and non-target trials.
3. Ensemble averages of 10, 20, 30, 40, 50, 60, 70, 80, 90, and 100 responses were formed by sampling without replacement from each of the respective categories.
4. A third order polynomial was fit to the ensemble average, as this is capable of capturing the shape of the target response.
5. Steps 3-4 were repeated 1000 times.
6. To test the model, the same approach was used as above, except data from the walking trials were used.

We found that the results could be classified with greater than 80% accuracy if ensembles of 20 trials were used, and greater than 90% accuracy if ensembles of 50 trials or more were used.


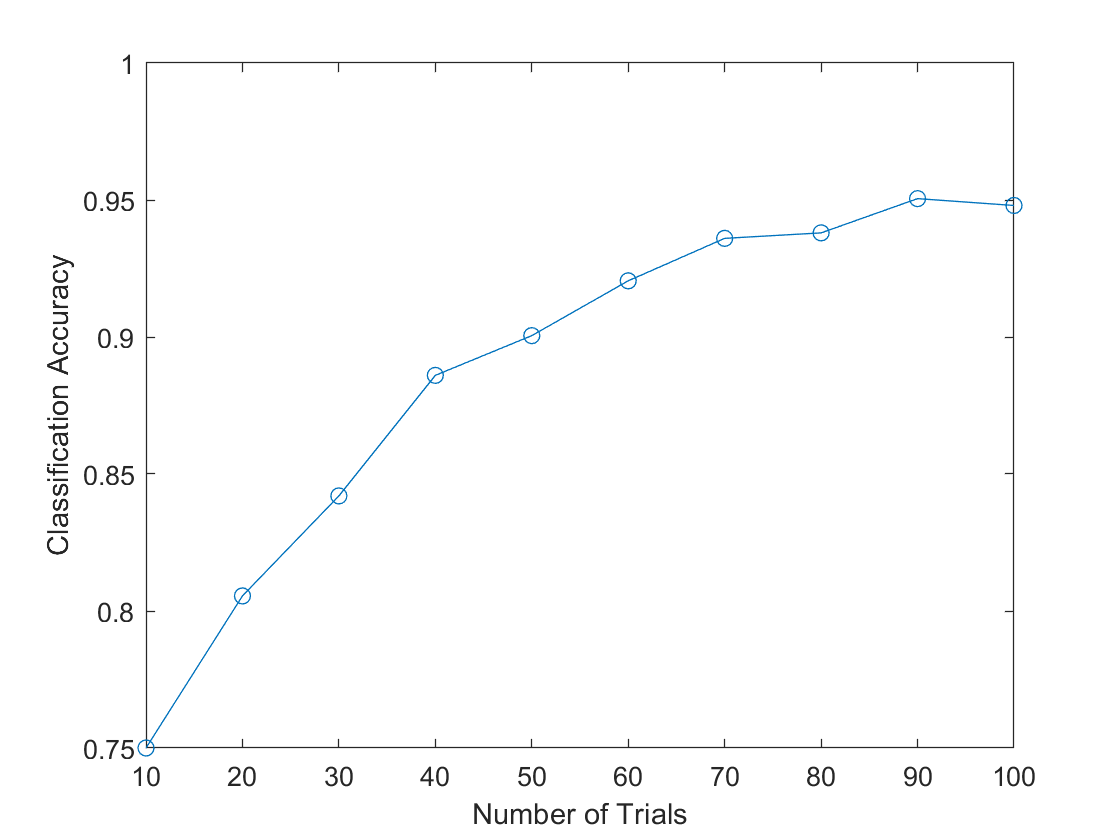


To show that the movement levels within the walking data did not impact the Pz amplitude, we also performed an additional classification analysis:

Head motion (rms)

Cluster

EEG voltage at Pz (μV)

Cluster

In the top plot, the x-axis shows the RMS for each trial in the walking data, grouped using k-means clustering. The x’s mark the centroids of each cluster.

In the bottom plot, the mean Pz amplitude is plotted against the same clusters that were assigned using the k-means clustering in the top plot. As you can see visually, there is no correlation between Pz amplitude and cluster number.

To quantify this classification, the left plot shows the true and prediced classes for each trial in the top plot above. The right plot shows the same but for the bottom plot above. These were classified using the built-in MATLAB function patternnet. Again, while there is a clear correlation between predicted class and true class in the left plot (which measures RMS motion and classes of 0.98), that is not the case for the right plot (which measures Pz amplitude and class with accuracy of 0.22). This suggests the motion level did not impact the Pz amplitude.

We can also see the SME as a measure of SNR. According to Luck et al., the SNR is better estimated by the SME. Here is our SME analysis:

SME of Targets:


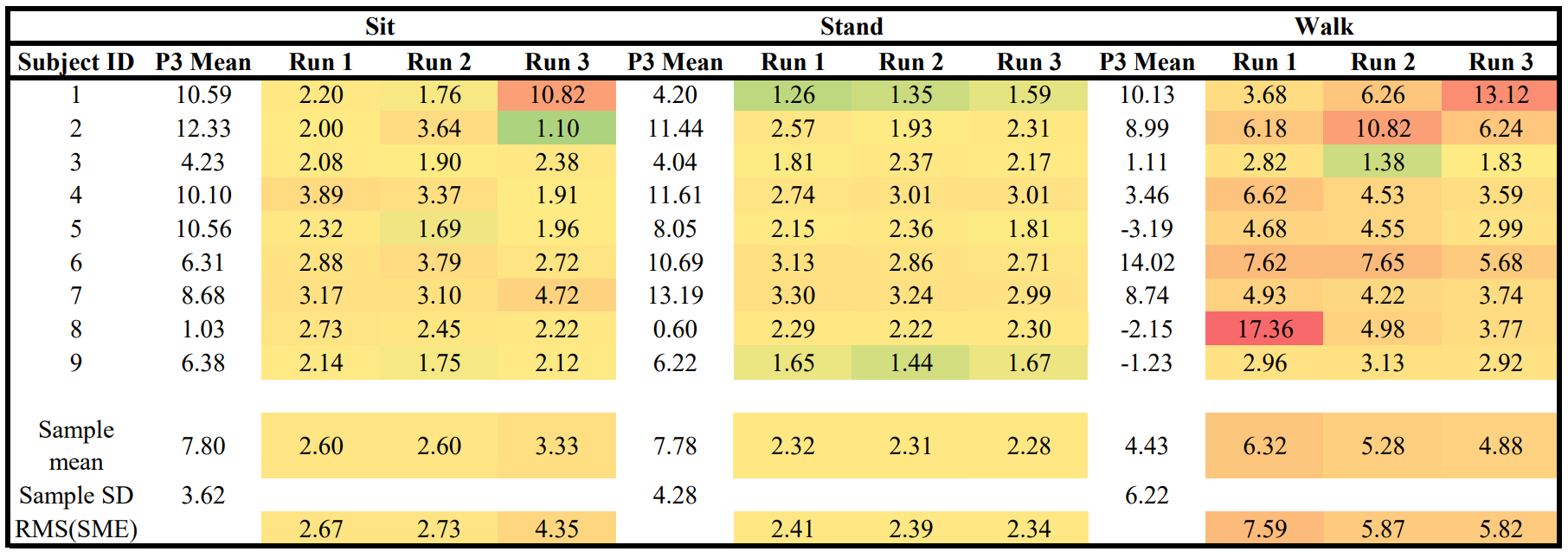


SME of Non-targets:


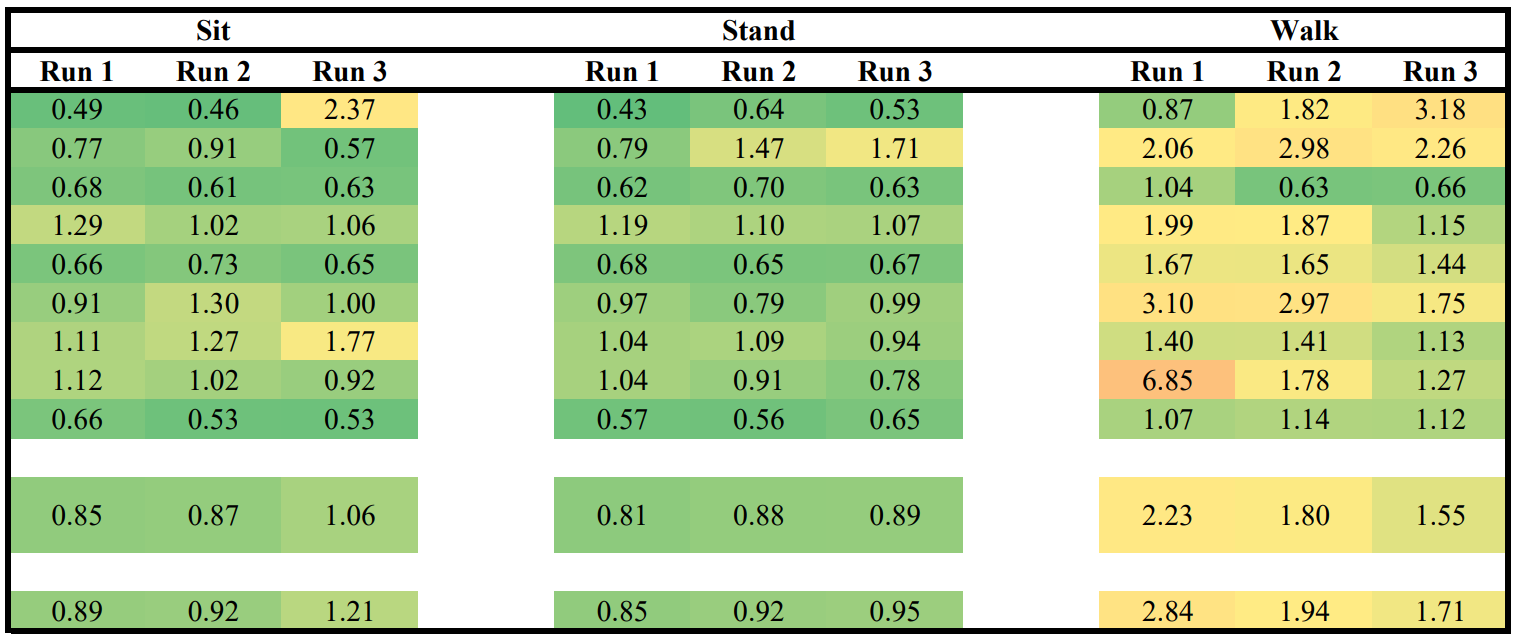


While this shows that the RMS(SME) is lower for sitting and standing trials, the walking trials are not that much different.

When comparing to the non-target trials, the walking trials seem on par between target and nontarget compared to the sitting and standing.

Luck SJ, Stewart AX, Simmons AM, Rhemtulla M. Standardized measurement error: A universal metric of data quality for averaged event‐related potentials. Psychophysiology. 2021 Jun;58(6):e13793.
